# Untargeted Urinary ^1^H NMR-Based Metabolomic Pattern as a Potential Platform in Breast Cancer Detection

**DOI:** 10.3390/metabo9110269

**Published:** 2019-11-07

**Authors:** Catarina L. Silva, Ana Olival, Rosa Perestrelo, Pedro Silva, Helena Tomás, José S. Câmara

**Affiliations:** 1 CQM—Centro de Química da Madeira, Universidade da Madeira, Campus Universitário da Penteada, 9020-105 Funchal, Portugal; cgsluis@gmail.com (C.L.S.); anaolival@uma.pt (A.O.); rmp@staff.uma.pt (R.P.); pedro_dasilva@hotmail.com (P.S.); lenat@staff.uma.pt (H.T.); 2 Faculdade de Ciências Exactas e Engenharia da Universidade da Madeira, Universidade da Madeira, Campus Universitário da Penteada, 9020-105 Funchal, Portugal

**Keywords:** breast cancer, ^1^H NMR, urine, metabolomics, chemometric tools

## Abstract

Breast cancer (BC) remains the second leading cause of death among women worldwide. An emerging approach based on the identification of endogenous metabolites (EMs) and the establishment of the metabolomic fingerprint of biological fluids constitutes a new frontier in medical diagnostics and a promising strategy to differentiate cancer patients from healthy individuals. In this work we aimed to establish the urinary metabolomic patterns from 40 BC patients and 38 healthy controls (CTL) using proton nuclear magnetic resonance spectroscopy (1H-NMR) as a powerful approach to identify a set of BC-specific metabolites which might be employed in the diagnosis of BC. Orthogonal partial least squares-discriminant analysis (OPLS-DA) was applied to a 1H-NMR processed data matrix. Metabolomic patterns distinguished BC from CTL urine samples, suggesting a unique metabolite profile for each investigated group. A total of 10 metabolites exhibited the highest contribution towards discriminating BC patients from healthy controls (variable importance in projection (VIP) >1, *p* < 0.05). The discrimination efficiency and accuracy of the urinary EMs were ascertained by receiver operating characteristic curve (ROC) analysis that allowed the identification of some metabolites with the highest sensitivities and specificities to discriminate BC patients from healthy controls (e.g. creatine, glycine, trimethylamine N-oxide, and serine). The metabolomic pathway analysis indicated several metabolism pathway disruptions, including amino acid and carbohydrate metabolisms, in BC patients, namely, glycine and butanoate metabolisms. The obtained results support the high throughput potential of NMR-based urinary metabolomics patterns in discriminating BC patients from CTL. Further investigations could unravel novel mechanistic insights into disease pathophysiology, monitor disease recurrence, and predict patient response towards therapy.

## 1. Introduction

The global cancer burden is estimated to have risen to 18.1 million new cases and 9.6 million deaths in 2018 (WHO) [1], being the second leading cause of death worldwide. Several genetic and epigenetic factors including ageing, unhealthy life styles (poor diet, tobacco and alcohol consumption), population growth, as well as the changing prevalence of certain causes of cancer linked to social and economic development, contribute for this sobering fact. Lung and female breast cancers are at the top of the leading cancer types in terms of the number of new cases, with approximately 2.1 million diagnoses in 2018, contributing about 11.6% of the total cancer incidence [1]. Furthermore, breast cancer is also the leading cause of cancer death in women (15.0%), followed by lung cancer (13.8%) and colorectal cancer (9.5%), which are also the second and third most common types of cancer, respectively [1]. Improving BC detection methods could reduce patient mortality and improve therapeutic responses and prognosis rates [2,3]. Common methods of routine surveillance for BC include periodic mammography, self- or physician performed examination, and blood tests of tumor markers, including cancer antigens (CA 15-3, CA 27.29), carcinoembryonic antigen (CEA), tissue polypeptide specific-antigen, and human epidermal growth factor receptor 2 (the shed form). CA 15-3 and CEA represent the most widely used tumor markers [4]. An additional factor that contributes to the poor prognosis of patients diagnosed with BC is the fact that the diagnosis is often delayed due to limitations in screening tests [5]. A recent approach is metabolomics that study a subset of small molecules derived from the global or targeted analysis of metabolic profiles from biological samples such as blood [6], urine [7,8,9,10], cells, or tissue [11,12,13,14,15], representing a valuable tool in the detection of several diseases including cancer. Several reports have already demonstrated the importance of studying the metabolome as means to discover a set of metabolites to be used as cancer biomarkers [16,17,18]. The study of specific metabolites to identify cancer fingerprints or signatures can aid in cancer detection and prognosis as well as the assessment of the pharmacodynamic effects of therapy [11]. The most common approaches in metabolomics involve gas chromatography-mass spectrometry (GC-MS) [8], liquid chromatography-mass spectrometry (LC-MS) [19] or nuclear magnetic resonance spectroscopy (NMR) [3,16,18,20].

Mass spectrometry (MS) includes a separation stage using liquid chromatography (LC) or gas chromatography (GC) and can discriminate between compounds based on a mass-to-charge (*m*/*z*) ratio in charged particles. Although compared with NMR, MS exhibits a greater sensitivity, sample preparation is laborious and dependent on metabolite chemical properties [21]. In addition, MS lacks accuracy and precision, producing an enhanced resolution profile with numerous peaks. However, only approximately 5% of these peaks are associated with known components [22]. Recently, non-invasive sampling strategies, including exhaled breath, urine and saliva, have emerged as attractive and useful approaches when coupled to high-throughput techniques such as NMR (^1^H NMR) [12,23,24]. NMR spectroscopy is a particularly appealing platform based on the energy absorption and re-emission of the nuclei due to the magnetic field, where the selected isotopes, having momentum in an external magnetic field, will give a signal. This technique presents several advantages such as being rapid, robust, cost-effective, highly reproducible, non-destructive, and fully quantitative. In addition, it requires no prior compound separation or derivatization generating spectra from a biological sample within few minutes, being useful for conducting metabolomic studies on biofluids [25,26,27]. Zhang et al. [28] reviewed the most common methods for NMR spectroscopy-based metabolite profiling, data processing and analysis. Bingol et al. [29] recently overview some advances in the metabolomics field and contribution to targeted and untargeted approaches. A variety of studies have been conducted by NMR in disease research using urine [3,12], saliva [24,30], serum [31], plasma [32,33], tissue [34,35,36,37], and in cell culture [38,39,40].

The aim of this study was to evaluate the ability of ^1^H NMR spectroscopy combined with multivariate statistical tools to differentiate and discriminate the urinary metabolomic patterns from BC patients and healthy volunteers (CTL) as a powerful approach to identify a set of BC-specific metabolites which might be employed in the diagnosis of BC.

## 2. Materials and Methods

### 2.1. Reagents

3-(Trimethylsilyl)propionic-2,2,3,3-d4 acid sodium salt (TSP) and deuterium oxide (D_2_O) were supplied by Acros Organics (Geel, Belgium). Potassium dihydrogen phosphate (KH_2_PO_4_), sodium azide and potassium deuteroxide solution (KOD) were purchased from Panreac (Barcelona, Spain) and Sigma Aldrich (St. Louis, MO, USA).

### 2.2. Urine Collection

To investigate the urinary NMR profile, 40 urine samples from female patients with BC (*n *= 40, age range 40–74 years, average 59 ± 10 years) and 38 urine samples from healthy female volunteers without any known pathology (*n *= 38, age range 40–72 years, average 53 ± 8 years; control group—CTL) were obtained from the Haemato-Oncology Unit of Hospital Dr. Nélio Mendonça (Funchal, Portugal) and Blood Donors Bank of the same Hospital, respectively (Table 1), and were randomly selected among the volunteers. Patients did not receive any neoadjuvant chemotherapy or radiation therapy prior to sample collection. Healthy controls were age- and gender-matched patients and had no declared history of cancer or gastrointestinal symptoms. Exclusion criteria included pregnancy, inflammatory conditions, mental disorders, gastrointestinal tract disorders, hypertension, uncontrolled bacterial, viral, or fungal infection and diabetes mellitus.

The study was approved by the Ethic Committee of Hospital Dr. Nélio Mendonça (Approval no. S.1708625/2017). Written informed consent for the study was obtained from all participants. Each individual (either as a patient or healthy volunteer) provided a sample of morning urine (after overnight fasting) in a 20 mL sterile container. The samples were aliquoted into 4 mL glass vials and frozen at –80 °C until the experiments.

Urine samples were thawed and centrifuged (8000 rpm for 5 min) to remove any suspended cells and other precipitated material [12]. Then, 540 µL of urine was mixed with 60 µL of a buffer solution (KH_2_PO_4_, 1.5 M in D_2_O) containing 0.1% of TSP-*d*_4_ (used as chemical shift reference) and sodium azide (NaN_3_, 2 mM). The pH was adjusted to 7.00 ± 0.02 by adding small amounts of KOD.

### 2.3. NMR Measurements

NMR spectral acquisition was performed using a Bruker Advance II Plus NMR spectrometer equipped with a 400 MHz magnet UltraShield™ 400 Plus at 300K. All NMR spectra acquisition and pre-processing were performed under the control of a workstation with TopSpin 3.1 (Bruker BioSpin). Two different ^1^H-NMR spectra were collected: a 1D ^1^H spectrum providing quantitative metabolite data for statistical analysis while 2D HSQC and 2D-Jres experiments assisted in peak assignment and metabolite identification using standard Bruker pulse programs. For each sample, a 1D nuclear overhauser enhancement spectroscopy (NOESY) pulse sequence (noesypr1d) was used in all cases and solvent signal suppression was achieved by presaturation during relaxation and mixing time (SW 4807.692 Hz, TD 64 K data points, relaxation delay 5 s, 128 scans). The shimming was calibrated automatically. Also, all spectra were processed using a line broadening (1.0 Hz) and baseline automatically corrected. The NMR spectrum of each sample was aligned with reference to the TSP signal at δ 0.00 ppm. Spectral regions within the range of 0.94 to 10 ppm were analyzed after excluding the sub-region δ 4.55–6.05 ppm to remove variability arising from water suppression and possible cross-relaxation effect on the urea signal via solvent exchanging protons. As already known, the TSP signal may be affected by proteins or other macromolecules present in samples [41] and for that reason in the preparation of urine samples before NMR analysis, the step of centrifugation was taken into account and the rotations per minute (rpm) was used in order to remove any proteins present in samples

The identification of metabolites was accomplished using the Chenomx NMR Suite 8.2 (Chenomx Inc., Alberta, Canada) and relative concentrations (in mM) of metabolites were determined using the 400MHz library from Chenomx NMR Suite 8.2, which compares the integral of a known reference signal (TSP) with signals derived from a library of compounds containing chemical shifts and peak multiplicities. In addition, the identification of selected metabolites was also cross checked from the Human Metabolome Database (HMDB) [42] and literature [43]. Regarding the metabolites that were not available in the library, identification was accomplished by running a standard solution and the relative concentration was calculated manually. This software not only allows the identification of compounds but also access their quantification based on advanced algorithms turning into a very straightforward tool to analyze NMR spectra.

### 2.4. Statistical Analysis

Statistical analyses were performed using the web server Metaboanalyst 3.0 [44] where sample specific normalization allowed the manual adjustment of relative concentrations based on biological inputs (i.e., volume, mass) and row-wise normalization allowed the general-purpose adjustment for differences among samples. Regarding data transformation and scaling were accomplished using two different approaches to make features more comparable, raw data were scaled using mean-centering and cubic root transformation. Intensities in each spectrum were normalized by the sum to avoid the contribution of urine dilution. Then, multivariate statistical analyses, namely, PCA, PLS-DA, and OPLS-DA were applied to the urinary metabolomic profile dataset to provide insights into the separations between the groups. Furthermore, hierarchical cluster analysis by k-means of the 2 groups in the study was performed and Pearson’s correlation was used to generate the heat map using the metabolites to identify clustering patterns. Moreover, the ROC curves were attained to verify which metabolites had the highest sensitivity/specificity for a BC diagnosis. Finally, the metabolites were used for the metabolic pathway analysis to identify the most relevant metabolic pathways involved in the BC and CTL groups.

## 3. Results and Discussion

### 3.1. Urinary Metabolomic Pattern Based on ^1^H NMR

**Figure 1:**
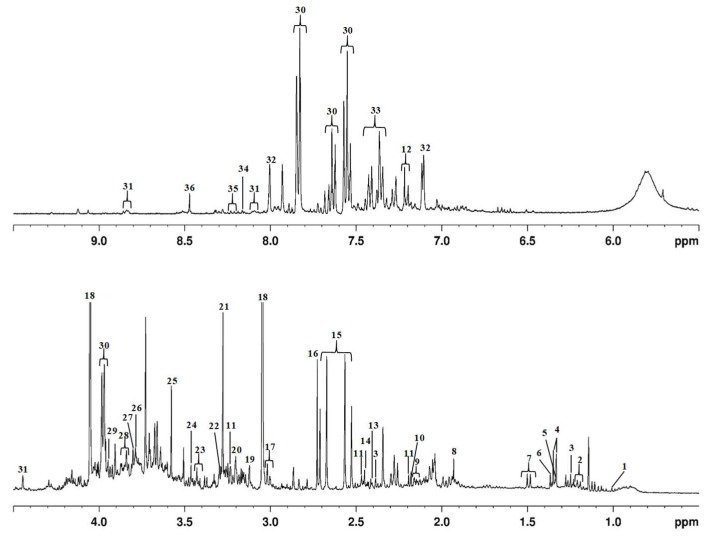
Typical 400 MHz representative urine ^1^H NMR spectrum from a BC patient, referenced to TSP (δ 0.00 ppm). For peak identification please see Table 2.

^1^H NMR analysis was performed according to the procedure described in the Methods section. A representative first dimension urine ^1^H NMR spectrum, referenced to TSP (δ 0. 0 ppm), from a BC patient is shown in Figure 1, and metabolites were indicated based on their chemical shifts.

Table 2 represents the identification of metabolites as well as their minimum and maximum relative concentrations (mM) for each group and the respective percentage of occurrence (FO). Each sample analysis was performed in triplicate and the relative standard deviation (RSD) was lower than 2%.

For most metabolites, the FO was higher than 90% with the following exceptions: valine, glutamine, carnitine, trigonelline, 4-cresol sulphate, and hypoxanthine for the BC group; α-hydroxyisobutyrate, trimethylamine N-oxide, hypoxanthine, and glycine for the CTL group. Regarding relative concentrations (in mM), the highest level was obtained for creatinine followed by hippurate in the BC group and citrate in the CTL group. In addition, taurine and mannitol presented superior levels in BC group, respectively. It can also be highlighted that the majority of metabolites were down-regulated relatively to the BC group, except formate, α-hydroxybutyrate hippurate, and phenylalanine, that were up-regulated, being also identified in a study developed by Carrola et al. [12] with lung cancer patients

Thirty-six metabolites were identified and quantified relative to TSP (Figure 1). The main metabolites identified in urine resulted mainly from tricarboxylate (e.g., citrate, *cis*-aconitate), methane (e.g., dimethylamine, trimethylamine *N*-oxide) and amino acid metabolisms (e.g., hippurate, glycine). The most intense signals were obtained from creatinine, creatine, hippurate, citrate, and trimethylamine *N*-oxide (Figure 1). These metabolites were already identified in several studies that use urine from cancer individuals [12,37,45]. Trimethylamine *N*-oxide is produced in the liver by intestinal bacteria from dietary quaternary amines, such as choline and carnitine trough trimethylamine (TMA) via flavin-containing monooxygenase (FMO3), and the levels in urine or plasma are used to determine FMO_3_ deficiency [46,47,48,49,50]. Creatinine is subsequently produced via a biological system involving creatine, phosphocreatine, and adenosine triphosphate (ATP), whereas hippurate and citrate are derived from phenylalanine metabolism and the citrate cycle [51]. The concentration of creatinine is age- and sex dependent, decreasing with age and varying throughout the day. Normally, the concentration of creatinine is increased in males when compared with females, given the increased body mass index [52]. Creatinine production from the muscles is proportional to the total muscle mass and muscle catabolism. In individuals with a relatively low muscle mass, including children, women, and cancer patients, serum creatinine levels are reduced for a given glomerular filtration rate (GFR), which is the flow rate of filtered fluid through the kidney, thus providing information on kidney function [53].

### 3.2. Multivariate Statistical Analysis of Urinary Metabolomic Profile

**Figure 2:**
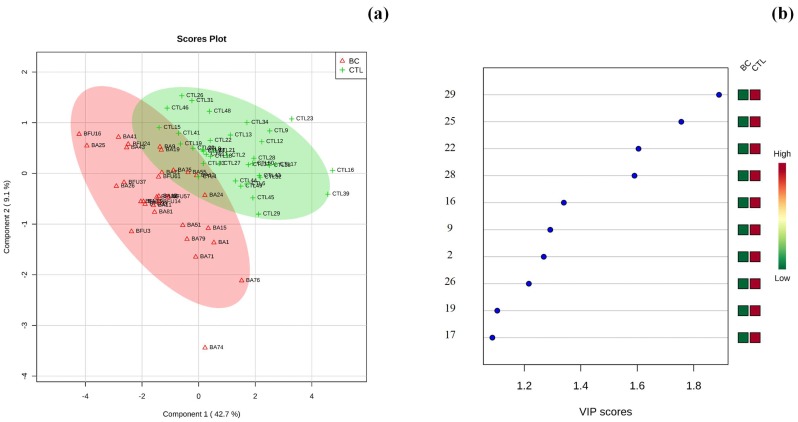
(**a**) Loading score plots of partial least square discriminant analysis (PLS-DA), and (**b**) VIP values of metabolites obtained by ^1^H NMR analysis of urine samples from the two groups in study. For number identification, see Table 2.

The first step before performing multivariate statistical analysis was to verify the normal distribution of the urinary metabolomic profile dataset using the Kolmogorov–Smirnov test (Table 2). All samples under analysis exhibited a normal distribution within each assigned group (*p *> 0.05). The samples under analysis that exhibited a normal distribution within each assigned group (those with *p*-values > 0.05 in the K–S column) were tested using the *t*-test to compare the means of the two groups (BC and CTL). For the samples that rejected the normality assumption (*p*-values < 0.05), the Mann–Whitney–Wilcoxon test was applied. The corresponding *p*-values associated with these tests are presented in a mean comparison column in Table 2. Furthermore, to obtain a reliable dataset to apply multivariate analysis, the dataset was evaluated to exclude the metabolites that had an FO < 90%. The dataset used to perform the statistical analysis also excluded creatinine, as mentioned above, as its concentration is dependent on age, gender, and disease status, decreasing with age and varying throughout the day. Regarding creatinine, relative concentrations obtained, and their respective differences between groups under study, this metabolite might be considered a potential artefact given that creatinine values may be altered, as the generation of creatinine may not be simply a product of muscle mass but influenced by muscle function, muscle composition, activity, diet, and health status [54]. Based on this, the dataset composed of 33 metabolites and 70 samples (32 BC and 38 CTL) that fulfilled this condition was subjected to principal component analysis (PCA). PCA as an unsupervised method was performed to visualize the similarities/differences between urine sample profiles of groups in this study. In this step, the samples were analyzed individually, e.g. without classification, according to the groups. A PCA score plot and loading plot from urine samples are presented in Supplementary Figure S1a,b. Although the projection of the variance between samples was performed without classification, it is possible to observe that the PCA of urine samples from BC patients and those from CTL presented a tendency for the formation of two clusters across the first principal component (PC1) that explains 54.6 % of the total variance. Most of the metabolites exhibited enormous importance in the variance projection of samples. Then, the partial least square-discriminant analysis (PLS-DA) was used as a supervised clustering method to maximize the separation between the groups and demonstrated that the samples tended to be grouped according with health condition of subject (BC and CTL) through its variance/covariance along the first component. Ten differently expressed metabolites that exhibited a variable importance in a projection (VIP) score greater than 1 were identified: creatine, glycine, serine, dimethylamine, trimethylamine N-oxide, α-hydroxyisobutyrate, mannitol, glutamine, cis-aconitate, and trigonelline (Figure 2a–d).

Many of these metabolites were already identified in various cancer types, including lung [12,55], breast, ovarian [3,5], bladder [56], and gastric cancers [57], in previous reports. Additionally, Zhou et al. [58] performed a metabonomics study using serum and urine from BC patients based on NMR, where citrate, phenylacetylglycine, and guanidoacetate exhibited significance in the discrimination of BC patients from healthy volunteers. In addition, Slupsky et al. [3] accomplished a study using urine from breast and ovarian cancer patients to discover metabolites for an early diagnosis. The authors found that certain intermediates of the tricarboxylic acid cycle and metabolites relating to energy metabolism, amino acids, and gut microbial metabolism, were perturbed. With regard to amino acids as raw materials of protein synthesis and catabolism products in vivo, their changes, whether in composition and concentration, can reflect the metabolic status of patients [58]. In addition, Cala et al. [59] established that the urinary and lipid profiles of Hispanic women also identified some of the same metabolites of this study, namely amino acids (valine, alanine, glycine, threonine), their levels being decreased in BC patients when compared to controls. This might be related to the requirement of amino acids in cancer metabolism in order to facilitate proliferation and cancer progression [60].

**Figure 3:**
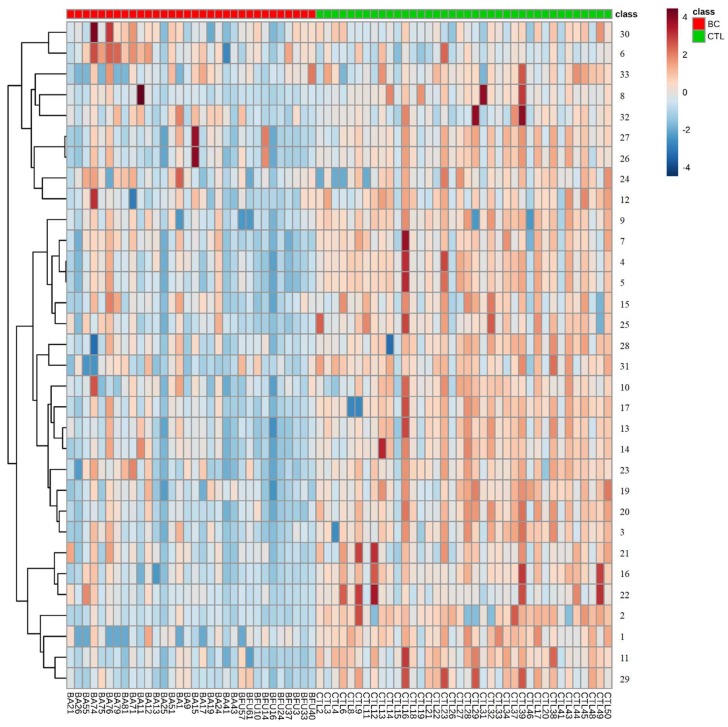
Heat map visualization and hierarchical clustering analysis by Pearson’s distance analysis.

Moreover, a heat map was constructed using Pearson’s correlation, providing intuitive visualization of the data set. The heat map contained the metabolites and was used to identify samples or features that are unusually high or low (Figure 3). As noted in Figure 3, the higher relative concentrations for most metabolites were found in the CTL group whereas the lowest relative concentrations were noted in the BC group.

Also, a heat map was generated for the dataset using Pearson’s correlation, providing an immediate visualization of data and possible correlations between samples (Figure 3).

**Figure 4:**
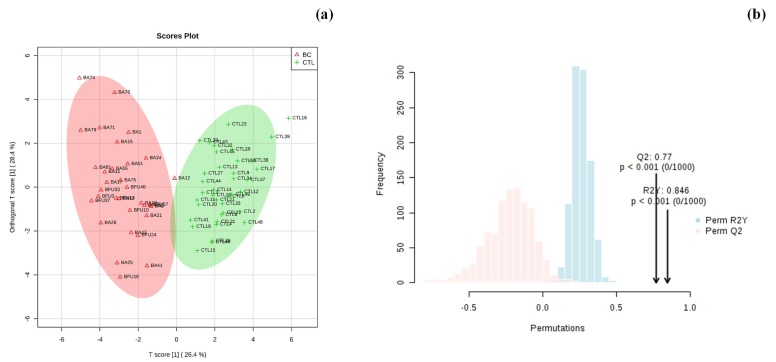
(**a**) Loading score plots of orthogonal projection to latent structure discriminant analysis (OPLS-DA), and (**b**) model validation by the permutation test based on 1000 permutations of metabolites obtained by 1H NMR analysis of urine samples from the two groups under study. The *p*-value based on permutation was *p* < 0.001 (0/1000).

Additionally, orthogonal partial least square-discriminant (OPLS-DA) analysis was applied to the urinary metabolomic profile dataset to maximize the separation between the CTL and BC groups. Figure 4a,b presents the scores and the loading plots for the OPLS-DA analysis, where it can be observed that a good separation was achieved with 54.8 % of total variance.

The OPLS-DA tool provides insights into the separation between the groups, demonstrating which variables are responsible for class discrimination. The robustness of the model was tested using a random permutation test with 1000 permutations (Figure 4b). This test yielded an R^2^ (that represents the goodness of fit) of 0.846 and a Q^2^ (that represents the predictive ability) of 0.770, indicating that the model is not over fitted and has a good predictive ability to distinguish between the groups under study.

**Figure 5:**
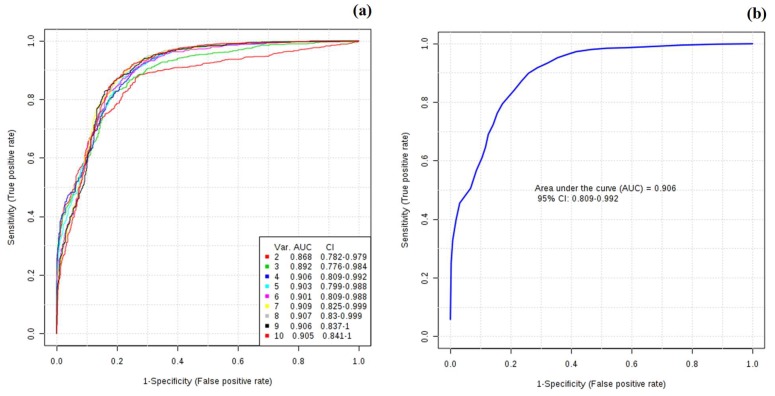
Receiver operating characteristic (ROC) curves for the predictive model. (**a**) A combination model calculated from the logistic regression analysis using the 10 metabolites selected by the VIP (>1.0) values, (**b**) ROC curves for the top 4 metabolites (creatine, glycine, trimethylamine N-oxide, and serine) with the highest ability to discriminate BC patients against CTL.

Moreover, receiver operating characteristic curves (ROCs) were generated for the two groups (CTL-BC) using the 10 identified metabolites with VIP values higher than 1 and are presented in Figure 5a,b.

As noted in the figure, as the number of metabolites increases, the area under the curve (AUC) also increases. Thus, using only 4 metabolites, the AUC value obtained was 0.91 for the CTL–BC, demonstrating the higher sensitivity/specificity to distinguish the groups. The metabolites with significance were creatine, glycine, serine, and trimethylamine N-oxide. These results are in accordance with the literature, where Xia et al. [61] report that an AUC value between 0.9 and 1.0 is excellent, and a value between 0.8 and 0.9 is good. By comparing the results, the values obtained were very good. A greater AUC value indicates a greater ability to distinguish the CTL from the BC group. The AUC can be interpreted as the probability that a randomly selected diseased subject is classified as diseased than a casually selected healthy subject [61].

Moreover, a 10-fold cross validation was used to generate a logistic regression model and the performance was calculated according to the equation below.

logit(P) = log(P/(1 − P)) = 0.471 + 1.434 creatine + 1.327 glycine + 0.25 serine + 0.285 trimethylamine N-oxide

where *P* is Pr(y = 1|x). The best threshold (or cutoff) for the predicted *P* was 0.39 (Tables S1 and S2).

**Figure 6:**
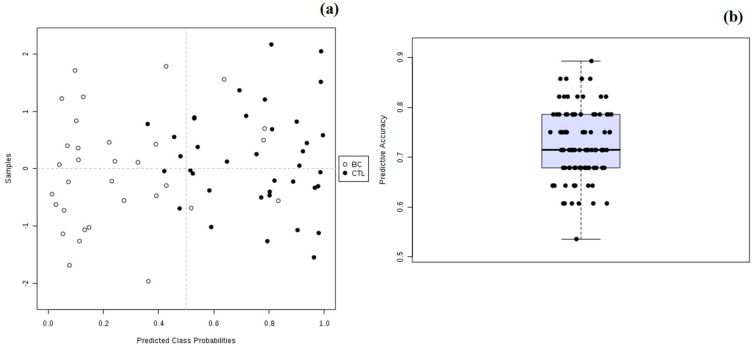
(**a**) Plot of the predicted class probabilities for all samples using the OPLS-DA biomarker model based on AUC and (**b**) box plot of the predictive accuracy (with an average of 0.910) of the biomarker model based on 100 cross validations.

Figure 6a,b show the results obtained for the predicted probabilities using the OPLS-DA model and the average of the predictive accuracy for the same model, where it can be observed that the model allowed good classification of samples (>90%). Moreover, 14 samples without known labels were processed together with the ones with known labels in order to obtain the probability of class labels, as presented in Table S3.

Most of the cases were classified in their respective group except for CTL49, BA71 and BA1, with a probability score ranging from 0.729 to 0.829 (Table S3).

Finally, the metabolic pathway analysis was performed to determine which pathways were altered in the groups under study.

**Figure 7:**
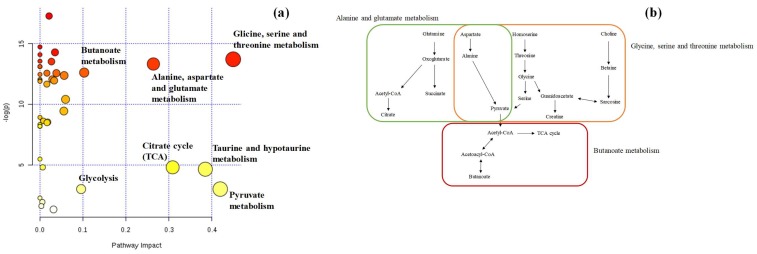
(**a**) The metabolome view map of significant altered metabolic pathways observed in urine from BC and CTL groups, and (**b**) metabolic pathways (*x*-axis) with highest impact that include the most promising potential BC biomarkers identified in this study.

Figure 7a,b presents the impacted pathways in the CTL-BC groups, respectively. The most impacted metabolic pathways were the glycine metabolism, the glutamate metabolism, the butanoate metabolism, glycolysis, the citrate cycle (TCA cycle), the taurine metabolism and the pyruvate metabolism, indicated by the red and yellow colors. It can be also highlighted that the pathway with the highest impact was the glycine metabolism (Figure 6b). The alterations are related to the down-regulation of the tricarboxylic acid (TCA) cycle (e.g., the Warburg effect) and the increased energy request in tumors [62]. It is well known that cancer cells convert more glucose into lactic acid than normal cells even in aerobic conditions, leading to a disturbance of the TCA cycle and their intermediates.

Based on these results, a successful differentiation and discrimination of samples was achieved between the CTL and BC groups. The results indicate that the ^1^H NMR urinary profile represents a useful approach to identifying potential BC biomarkers.

## 4. Conclusions

This study assessed the metabolomic urinary profile of BC patients in active and follow-up stages compared with that in healthy volunteers, using ^1^H NMR combined with multivariate statistical tools (PCA, PLS-DA, and OPLS-DA) that were applied to two groups (BC and CTL). Thirty-three metabolites were identified and quantified using Chenomx software. Multivariate statistical analysis revealed some metabolites were significantly altered in BC patients. Of the metabolites detected, creatine, glycine, serine, dimethylamine, trimethylamine N-oxide, α-hydroxyisobutyrate, mannitol, glutamine, cis-aconitate, and trigonelline exhibited the highest sensitivities and specificities to discriminate BC patients from healthy controls. A plot analysis revealed a metabolomic biosignature comprising an array of several biochemical pathways altered in BC patients. A metabolic pathway analysis indicated that the discriminatory metabolites potentially originated from several dysregulated pathways in BC: the glycine metabolism, the glutamate metabolism, the butanoate metabolism, glycolysis, the citrate cycle (TCA cycle), the taurine metabolism and the pyruvate metabolism. Moreover, although this was a small sample cohort without discrimination against BC subtypes, the results obtained were promising, indicating the usefulness of endogenous metabolites for biomarker discovery metabolites and the need to investigate the related metabolomic pathways in order to improve the diagnostic tools of BC.

## Figures and Tables

**Table 1:** List of collected urine samples from breast cancer patients and healthy individuals (controls).

Sample Group	N° Urine Samples	Age Range/Years	Mean Age ± SD ^1^
Breast Cancer (BC)	*n* = 40	40–74	59 ± 10
Control (CTL)	*n *= 38	40–72	53 ± 8

^1^ SD: standard deviation.

**Table 2:** Metabolites found in urine samples from breast cancer (BC) patients and healthy controls (CTL) (*n* = 3, % RSD <2).

Peak n°	Metabolite	δ (ppm)	Relative Concentrations (mM)						Variation	K-S ^3^ (Normality)					FO (%) ^6^	
			CTL			BC				CTL		BC		Mean Comparison ^5^	CTL	BC
		Multiplicity	Min ^1^	Max ^2^	Average	Min	Max	Average		Statistic ^4^	*p*-Value	Statistic	*p*-Value			
6	α-hydroxyisobutyrate	1.35 (s) ^7^	1.21	6.64	3.94	0.15	0.89	0.55	↓	0.118	0.200	0.138	0.200	1.10 × 10^−12^	78	100
18	creatinine	3.03 (s), 4.05 (s)	116.84	381.60	200.56	10.49	216.36	83.87	↓	0.231	0.200	0.265	0.103	9.23 × 10^−11^	100	100
13	pyruvate	2.36 (s)	1.90	4.35	3.34	0.47	3.71	1.86	↓	0.317	0.032	0.114	0.200	3.86 × 10^−10^	100	100
5	threonine	1.32 (d) ^8^, 3.58 (d), 4.25 (m) ^9^	2.15	6.81	4.33	0.74	4.09	2.36	↓	0.203	0.200	0.197	0.200	5.12 × 10^−10^	98	93
17	α-oxoglutarate	2.43 (t) ^10^, 2.99 (t)	3.00	10.54	6.33	0.82	6.13	3.07	↓	0.245	0.200	0.207	0.200	8.78 × 10^−10^	95	92
3	β-hydroxyisovalerate	1.26 (s), 2.35 (s)	0.71	1.64	1.19	0.15	1.16	0.57	↓	0.166	0.200	0.298	0.035	7.15 × 10^−9^	98	100
16	dimethylamine	2.72 (s)	3.43	9.71	7.00	0.31	8.07	3.61	↓	0.232	0.200	0.267	0.097	1.00 × 10^−8^	95	100
8	acetate	1.91 (s)	1.30	3.30	2.03	0.56	2.44	1.36	↓	0.202	0.200	0.206	0.200	1.00 × 10^−7^	95	96
4	lactate	1.32 (d), 4.11 (m)	2.14	6.41	3.99	0.75	4.71	2.88	↓	0.174	0.200	0.153	0.200	1.10 × 10-^7^	98	100
20	choline	3.19 (s), 3.51 (m), 4.06 (m)	0.93	2.45	1.61	0.25	2.07	1.08	↓	0.221	0.200	0.223	0.200	1.20 × 10^−7^	100	96
28	serine	3.84 (q) ^11^, 3.94 (q), 3.98 (q)	15.00	60.92	29.59	5.03	30.12	15.32	↓	0.231	0.200	0.220	0.200	3.59 × 10^−7^	95	92
11	carnitine	2.40 (s), 2.45 (s), 3.21 (s)	0.65	2.95	1.70	0.15	1.97	0.86	↓	0.218	0.200	0.200	0.200	1.46 × 10^−6^	100	80
14	succinate	2.39 (s)	0.70	1.61	1.04	0.18	1.25	0.68	↓	0.192	0.200	0.146	0.200	1.88 × 10^−6^	100	96
9	glutamine	2.10 (t), 2.42 (m), 3.77 (t)	1.47	11.19	7.13	1.65	10.81	4.78	↓	0.219	0.200	0.242	0.186	2.87 × 10^−6^	93	67
12	4-cresol sulphate	2.24 (s), 6.82 (m), 7.23 (m)	1.22	11.91	5.51	0.79	4.92	2.09	↓	0.216	0.200	0.191	0.200	1.38 × 10^−5^	100	84
36	formate	8.44 (s)	0.18	1.58	0.70	1.44	6.66	3.75	↑	0.256	0.184	0.349	0.005	1.54 × 10^−5^	77	79
29	creatine	3.03 (s), 3.92 (s)	6.19	40.74	20.77	1.50	17.00	7.46	↓	0.194	0.200	0.225	0.200	3.29 × 10^−5^	98	92
27	guanidoacetate	3.79 (s)	7.98	21.04	12.41	1.71	14.89	8.72	↓	0.206	0.200	0.171	0.200	5.38 × 10^−5^	100	92
19	cis-aconitate	3.11 (d), 5.72 (m)	4.08	12.31	7.51	0.59	12.20	5.14	↓	0.167	0.200	0.207	0.200	8.28 × 10^−5^	95	100
7	alanine	1.47 (d), 3.78 (m)	1.59	4.48	2.90	0.57	3.51	2.29	↓	0.202	0.200	0.255	0.136	8.42 × 10^−5^	100	100
1	valine	1.03 (d), 2.26 (m), 3.60 (d)^4^	0.32	1.14	0.60	0.27	1.24	0.50	↓	0.270	0.131	0.310	0.023	1.35 × 10^−4^	95	68
10	acetone	2.22 (s)	0.78	2.69	1.60	0.33	2.31	1.28	↓	0.167	0.200	0.171	0.200	1.64 × 10^−4^	100	92
22	trimethylamine N-oxide	3.25 (s)	3.37	15.65	8.96	1.31	18.04	5.63	↓	0.173	0.200	0.300	0.033	2.82 × 10^−4^	85	92
26	mannitol	3.67 (m), 3.75 (m), 3.79 (d)	14.24	49.04	28.85	3.67	24.04	16.10	↓	0.230	0.200	0.167	0.200	6.31 × 10^−4^	100	92
25	glycine	3.55 (s)	1.93	43.52	18.57	1.88	22.13	10.20	↓	0.166	0.200	0.166	0.200	9.26 × 10^−4^	88	100
31	trigonelline	4.43 (s), 8.07 (t), 8.83 (m), 8.78 (m)	1.36	4.21	2.79	0.39	6.13	2.68	↓	0.168	0.200	0.264	0.106	1.35 × 10^−3^	100	88
15	citrate	2.53 (d), 2.69 (d)	9.14	85.10	33.04	10.58	55.47	29.53	↓	0.327	0.023	0.231	0.200	2.10 × 10^−3^	100	100
23	taurine	3.25 (t), 3.42 (t)	6.66	12.95	10.18	1.93	14.46	6.80	↓	0.164	0.200	0.307	0.026	2.58 × 10^−3^	98	92
2	α-hydroxybutyrate	1.19 (d), 2.27 (m), 2.39 (m)	0.47	1.30	0.77	0.39	3.38	1.47	↑	0.208	0.200	0.318	0.017	2.71 × 10^−3^	100	95
21	betaine	3.25 (s), 3.89 (s)	0.52	2.62	1.37	0.22	1.96	1.05	↓	0.261	0.163	0.144	0.200	1.71 × 10^−2^	93	100
35	hypoxanthine	8.18 (s), 8.20 (s)	0.45	2.77	1.57	0.31	2.14	0.78	↓	0.214	0.200	0.316	0.018	3.42 × 10^−2^	80	64
34	3-methylhistidine	3.22 (m), 3.31 (m), 3.73 (s), 3.96 (q), 8.08 (s)	0.56	11.63	4.33	0.54	10.87	2.44	↓	0.224	0.200	0.419	0.000	1.74 × 10^−1^	93	100
24	4-hydroxyphenylacetate	3.44 (s), 6.85 (d), 7.15 (d)	0.90	4.14	1.89	0.59	5.02	1.57	↓	0.287	0.084	0.282	0.061	3.65 × 10^−1^	90	88
32	histidine	7.08 (m), 7.87 (d)	0.12	4.06	0.95	0.33	1.09	0.57	↓	0.406	0.001	0.206	0.200	3.88 × 10^−1^	88	89
33	phenylalanine	7.31 (m), 7.37 (m), 7.43 (m)	0.97	4.84	1.98	0.91	5.99	2.66	↑	0.252	0.200	0.182	0.200	6.93 × 10^−1^	76	88
30	hippurate	3.96 (d), 7.54 (t), 7.63 (t), 7.82 (d)	5.56	70.01	24.73	2.27	58.30	33.76	↑	0.258	0.174	0.313	0.021	7.48 × 10^−1^	93	96

*Legend:*^1^ Min—minimum relative concentration; ^2^ Max—maximum relative concentration; ^3^ K–S—Kolmogorov–Smirnov (normality test); ^4 ^observed value related to K–S test regarding normality; ^5^ Mean comparison using *t*-test or Mann–Whitney–Wilcoxon test; ^6^ frequency of occurrence;^ 7^ (s) singlet; ^8 ^(t) triplet; ^9^ (m) multiplet; ^10^ (d) duplet; ^11^ (q) quartet.

## Supplementary Materials

The following are available online at https://www.mdpi.com/2218-1989/9/11/269/s1, Figure S1: Scores plot (a) and loading plots (b) of PCA from urine samples based on 33 metabolites. For numbers identification see Table 2.
