# Supplementary figures and images for: Untargeted Urinary 1H NMR-Based Metabolomic Pattern as a Potential Platform in Breast Cancer Detection
Source: Metabolites. 2019 Nov 7;9(11):269. doi: 10.3390/metabo9110269 (PMC6918409; doi:10.3390/metabo9110269)

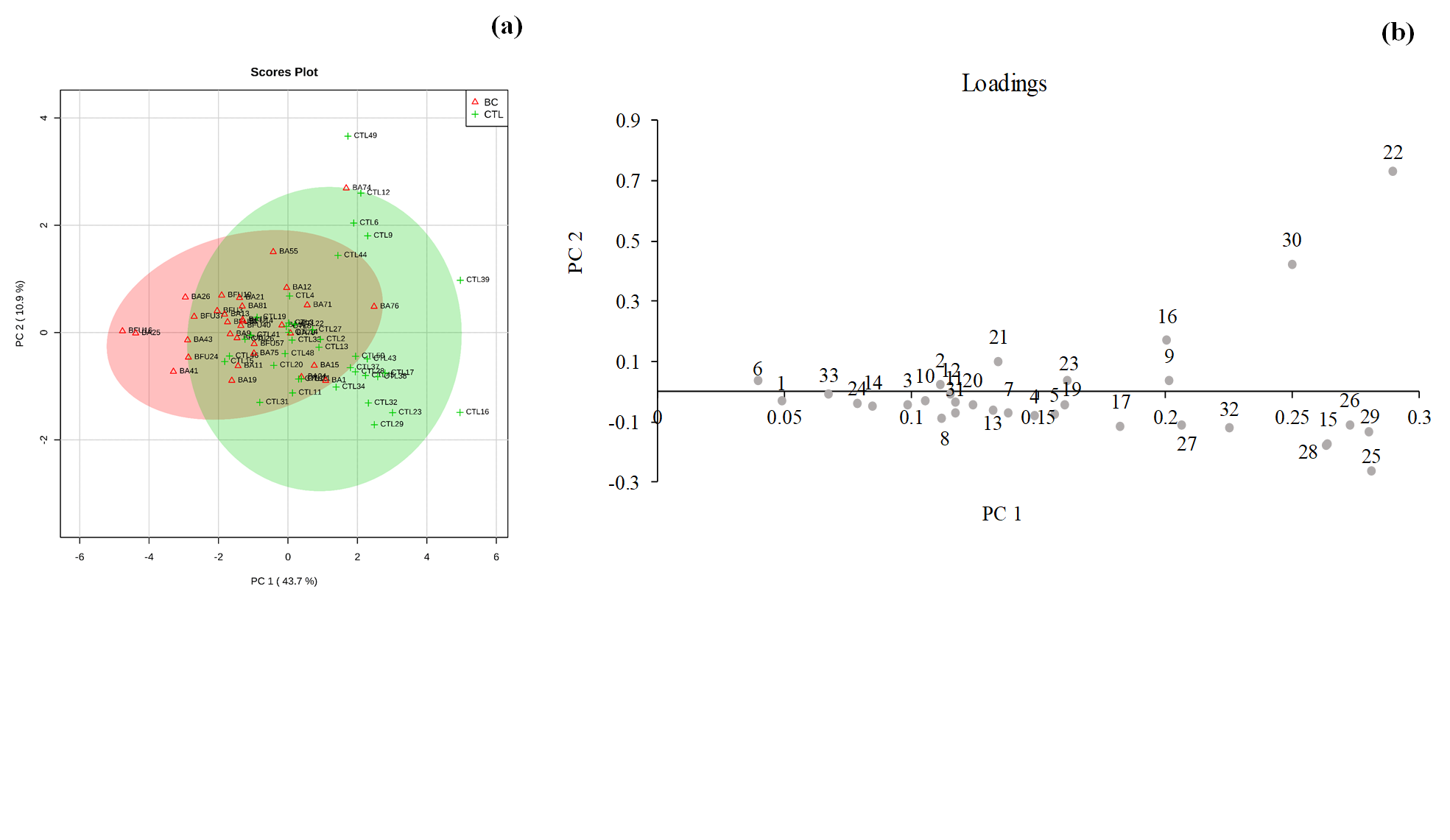

Supplement: Supplementary file 1 [file metabolites-09-00269-s001.zip › metabolites-627411-supplementary.tif]
